# Nuclear translocation of annexin 1 following oxygen-glucose deprivation–reperfusion induces apoptosis by regulating *Bid* expression via p53 binding

**DOI:** 10.1038/cddis.2016.259

**Published:** 2016-09-01

**Authors:** Xing Li, Yin Zhao, Qian Xia, Lu Zheng, Lu Liu, Baoming Zhao, Jing Shi

**Affiliations:** 1 Department of Neurobiology, Tongji Medical College, Huazhong University of Science and Technology, Wuhan, People's Republic of China; 2 Key Laboratory of Neurological Diseases, Ministry of Education, Wuhan, People's Republic of China; 3 Institute for Brain Research, Huazhong University of Science and Technology, Wuhan, People's Republic of China

## Abstract

Previous data have suggested that the nuclear translocation of annexin 1 (ANXA1) is involved in neuronal apoptosis after ischemic stroke. As the mechanism and function of ANXA1 nuclear migration remain unclear, it is important to clarify how ANXA1 performs its role as an apoptosis ‘regulator' in the nucleus. Here we report that importazole (IPZ), an importin *β* (Imp*β*)-specific inhibitor, decreased ANXA1 nuclear accumulation and reduced the rate of neuronal death induced by nuclear ANXA1 migration after oxygen-glucose deprivation–reoxygenation (OGD/R). Notably, ANXA1 interacted with the *Bid* (BH3-interacting-domain death agonist) promoter directly; however; this interaction could be partially blocked by the p53 inhibitor pifithrin-*α* (PFT-*α*). Accordingly, ANXA1 was shown to interact with p53 in the nucleus and this interaction was enhanced following OGD/R. A luciferase reporter assay revealed that ANXA1 was involved in the regulation of p53-mediated transcriptional activation after OGD/R. Consistent with this finding, the nuclear translocation of ANXA1 after OGD/R upregulated the expression of Bid, which was impeded by IPZ, *ANXA1* shRNA, or PFT-*α*. Finally, cell-survival testing demonstrated that silencing ANXA1 could improve the rate of cell survival and decrease the expression of both cleaved caspase-3 and cleaved poly(ADP-ribose) polymerase. These data suggested that Imp*β*-dependent nuclear ANXA1 migration participates in the OGD/R-dependent induction of neuronal apoptosis. ANXA1 interacts with p53 and promotes p53 transcriptional activity, which in turn regulates *Bid* expression. Silencing ANXA1 decreases the expression of Bid and suppresses caspase-3 pathway activation, thus improving cell survival after OGD/R. This study provides a novel mechanism whereby ANXA1 regulates apoptosis, suggesting the potential for a previously unidentified treatment strategy in minimizing apoptosis after OGD/R.

Ischemia–reperfusion is a well-recognized pathological condition that is characterized by an initial deprivation of blood supply to an area or organ followed by subsequent vascular restoration and concomitant reoxygenation of downstream tissues.^1, 2^ Ischemia–reperfusion occurs during various complications of vascular diseases, such as stroke and myocardial infarction.^1, 3, 4, 5^ Oxygen-glucose deprivation–reoxygenation (OGD/R) is an accepted model for studying ischemia–reperfusion *in vitro*.^6, 7, 8^ Previous findings have indicated that annexin 1 (ANXA1) is involved in neuronal apoptosis after OGD/R, but the underlying mechanism remains unclear.^9^ Therefore, it is important to clarity the regulatory role(s) of ANXA1 in OGD/R-mediated apoptosis.

As a Ca^2+^- and phospholipid-binding protein,^10, 11^ ANXA1 performs different roles depending on its subcellular localization. Kim *et al.*^12^ reported that phorbol 12-myristate 13-acetate (PMA)-induced ANXA1 nuclear translocation might participate in the regulation of cell proliferation and differentiation. Wang *et al.*^13^ revealed that kirenol and prednisolone promote the nuclear localization of ANXA1, which then interacts with NF-*κ*B to inhibit NF-*κ*B activity, reducing cytokine expression and thereby attenuating the inflammation of collagen-induced arthritis. Results from our previous study indicated that ANXA1 migration into the nucleus and its regulation of *Bid* (BH3-interacting-domain death agonist) gene expression were involved in apoptosis.^9^ However, exactly how ANXA1 regulates *Bid* expression and induces apoptosis after OGD/R remains unclear.

Bid can participate in neuronal apoptosis induced by brain ischemia,^14, 15^ and its expression is regulated by the transcription factor p53.^16, 17^ The genomic loci for both the human and the mouse *Bid* genes contain p53-binding, DNA-response elements that bind p53 and mediate p53-dependent transactivation of reporter genes.^18, 19^ Unlike all other known BH3-only proteins, Bid needs to be cleaved by caspase-8 or other proteases into tBid to become activated, whereupon it translocates to the mitochondrial outer membrane^20^ where it leads to the activation of Bax/Bak, thereby inducing the cytosolic release of cytochrome *c* from the mitochondria after ischemia.^21, 22^ In turn, the released cytochrome *c* ultimately leads to the cleavage and activation of caspase-9 and caspase-3,^23^ representing the final steps of the intrinsic caspase pathway that has been previously shown to regulate ischemic cell death.^15, 24^

The p53 tumor suppressor protein acts as a major defense mechanism against cancer.^25^ Among its most distinctive features is its ability to elicit both apoptotic death and cell cycle arrest.^26^ Numerous p53 co-factors have been implicated in cellular processes, and the activation or suppression of these co-factors determines the fate of a cell.^27, 28^ Whether ANXA1 represents one of the p53 co-factors involved in the regulation of *Bid* expression and how ANXA1 might interact with p53 to coregulate *Bid* expression have yet to be determined.

In this study, we investigated the role of ANXA1 in neuronal apoptosis after OGD/R. Specifically, we examined the requirement for importin *β* (Imp*β*)-activation in nuclear migration of ANXA1 and determined the interaction and coregulatory status of ANXA1 and p53 in the cell nucleus with respect to p53 transcriptional activity, *Bid* expression, and eventual caspase-3/poly(ADP-ribose) polymerase (PARP) activation after OGD/R.

## Results

### Nuclear ANXA1 translocation mediated by functional Imp*β* induces neuronal death after OGD/R

**Figure 1:**
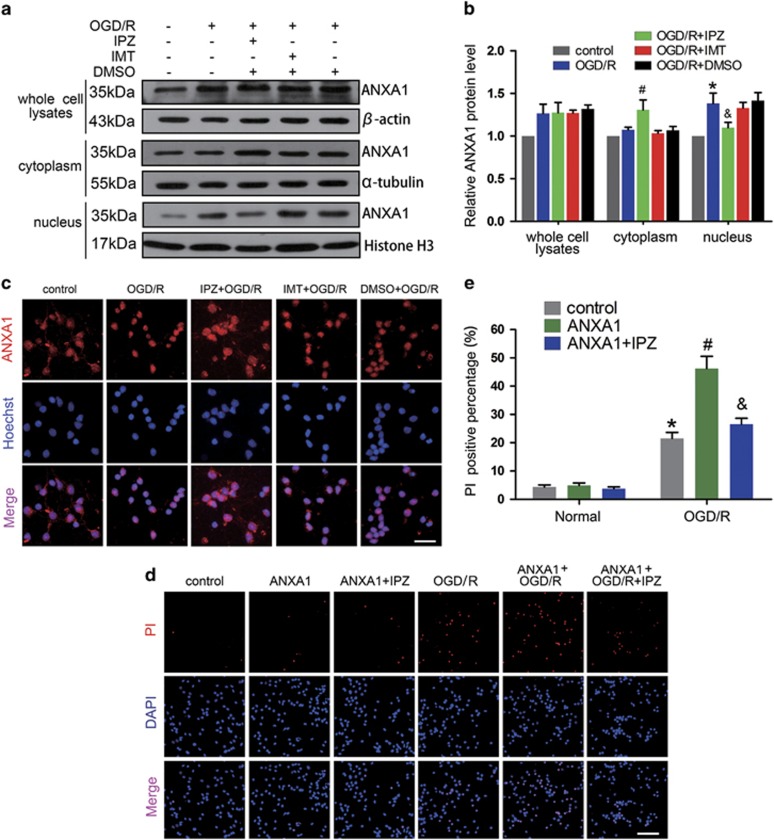
Expression of ANXA1 and neuronal death after OGD/R. (**a**) Western blots showing ANXA1 expression in primary cultured neurons treated with IPZ or IMT after OGD/R. (**b**) Statistical analysis of the data shown in (**a**). The data shown are expressed as the means±S.E.M. from three independent experiments. *P<0.05 *versus* control, ^#^*P*<0.05 *versus* OGD/R and ^&^*P*<0.05 *versus* OGD/R. (**c**) Representative images of ANXA1 expression in primary cultured neurons treated with IPZ or IMT after OGD/R. Scale bar=25 *μ*m. (**d** and **e**) Representative PI staining and statistical analysis show the effect of ANXA1 overexpression on cell death in primary cultured neurons after OGD/R. Cell death was visualized by PI staining. Scale bar=50 *μ*m. The data are expressed as the means±S.E.M. from three independent experiments. *P<0.05 *versus* control, ^#^*P*<0.05 *versus* OGD/R and ^&^*P*<0.05 *versus* ANXA1+OGD/R

Imp*α* and -*β* are considered classic nuclear envelope transporters. We used the specific Imp*β* inhibitor importazole (IPZ)^29^ (8 *μ*M) and the specific Imp*α* inhibitor ivermectin (IMT)^30^ (25 *μ*M) to separately block the functions of Imp*β* and Imp*α*. To investigate the subcellular location of ANXA1 after OGD/R, we extracted cytoplasmic and nuclear proteins from primary cultured neurons. As shown in Figures 1a and b, OGD/R increased the expression of ANXA1 in whole-cell lysates. Furthermore, OGD/R increased ANXA1 nuclear translocation without effect on its expression in the cytoplasm. Treatment with IPZ, but not IMT, reduced ANXA1 expression in the nucleus. Immunofluorescence analysis also indicated that under normal conditions, ANXA1 primarily localized to the cytoplasm in neuronal cells and IPZ could inhibit OGD/R-induced nuclear ANXA1 translocation (Figure 1c). To determine the effect of ANXA1 on neuron death after OGD/R, the adenovirus vector carrying rat *ANXA1* gene was constructed and overexpression of ANXA1 in primary cultured neurons was confirmed by western blot (Supplementary Figures S1a–c). Then, we performed propidium iodide (PI) staining followed by *ANXA1* transfection, IPZ, or (and) OGD/R treatment. The results revealed that OGD/R-induced cell death and that overexpression of ANXA1 associated with OGD/R promoted neuron death. In contrast, treatment with IPZ markedly decreased nuclear ANXA1 translocation and subsequent cell death after OGD/R in neuron (Figures 1d and e). Taken together, these results suggested that nuclear ANXA1 migration depended upon functional Imp*β* and was involved in OGD/R-induced neuronal cells death.

### ANXA1 accumulates on the *Bid* gene promoter

**Figure 2:**
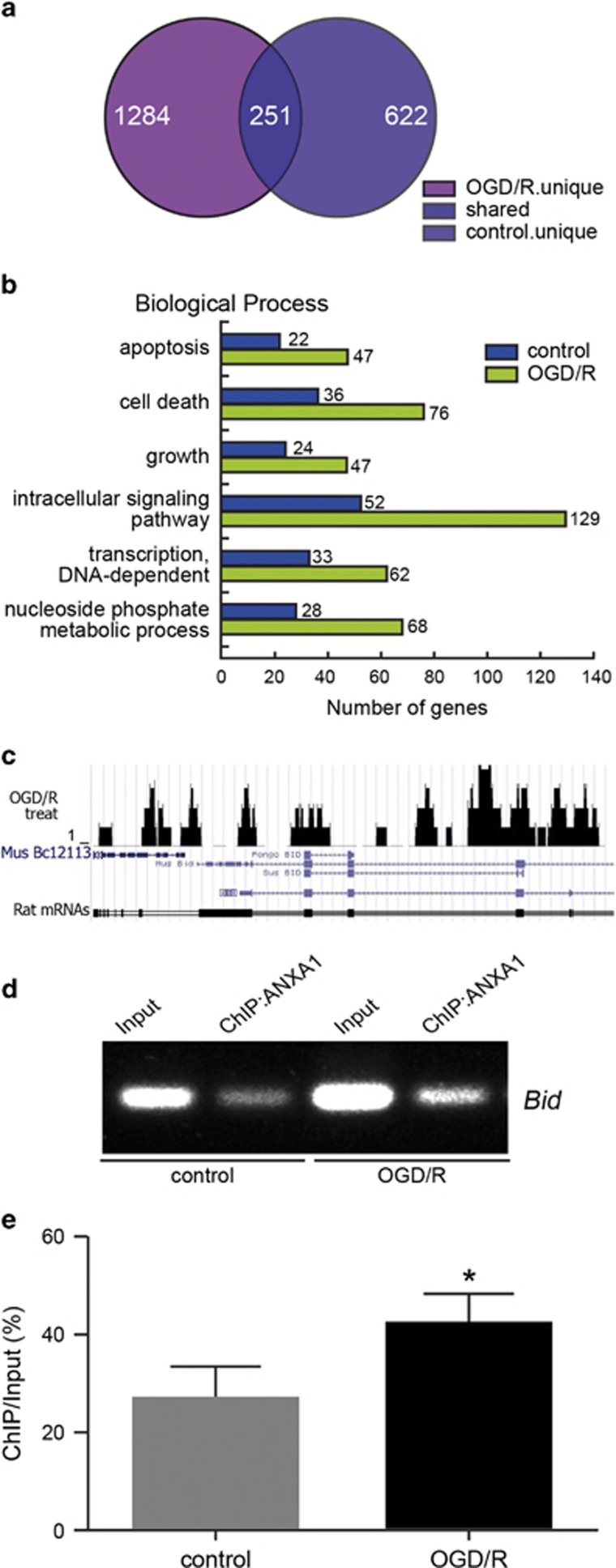
Effect of OGD/R on ANXA1 accumulation on the *Bid* gene promoter. (**a**) Venn diagram of the overlap among all ANXA1-bound genes identified by ChIP-Seq in primary cultured neurons treated without or with OGD/R. The genes that bound ANXA1 from the normal and OGD/R group (*n*=3 independent samples) are shown. (**b**) GO enrichment of ANXA1-bound proteins compared with the total genes from the rat genome. Significant enrichments of GO terms were obtained for the biological processes shown. (**c**) UCSC Genome Browser tracks depicting ANXA1 ChIP-Seq peaks at representative *Bid* gene loci under OGD/R conditions. (**d**) ChIP-PCR results show the interaction between ANXA1 and the *Bid* gene promoter after OGD/R in HEK293 cells. (**e**) Statistical analysis of the data shown in (**d**). The data are expressed as the means±S.E.M. from three independent experiments. *P<0.05 *versus* control

To determine the function of ANXA1 in the nucleus, and especially potential physical interactions with apoptosis-related gene loci, we performed chromatin immunoprecipitation (ChIP) assays to isolate ANXA1-DNA-binding complexes from primary cultured rat neurons, after which ChIP-Seq was performed on a Solexa Genome Analyzer (Solexa, Cambridge, UK) to identify the complex components in cooperation with BGI-Shenzhen (China). We identified 622 different genes that interacted with ANXA1 under normal physiological conditions and 1284 genes under OGD/R conditions, of which 251 genes were overlapping (Figure 2a). Gene ontology (GO) analyses were also used to explore the functional annotations of these target genes. These genes were classified into six categories, based on their involvement in different biological processes (Figure 2b). We found that ANXA1 potentially interacts with 22 different apoptosis-related genes and 36 cell death-related genes under normal conditions, and with 47 apoptosis-related genes and 76 cell death-related genes following OGD/R (Figure 2b). Comparison of the ChIP-Seq data with the UCSC Genome Browser for the rat genome (March 2012; RGSC 5.0/rn5) revealed that ANXA1 located to the *Bid* gene promoter domain following OGD/R (Figure 2c). To confirm these results, a ChIP-polymerase chain reaction (PCR) assay was performed. As shown in Figures 2d and e, ANXA1 directly associated with the *Bid* gene promoter under both normoxic and OGD/R conditions in human embryonic kidney 293 (HEK293) cells, although the total level of ANXA1 binding to the *Bid* promoter was increased after OGD/R.

### ANXA1 promotes the transcription and translation of Bid under OGD/R conditions

**Figure 3:**
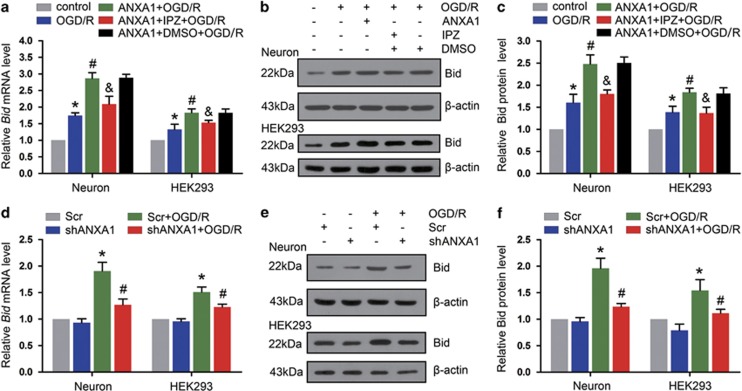
Effect of ANXA1 nuclear migration on *Bid* expression. (**a**) qPCR shows the effects of ANXA1 overexpression and IPZ treatment on *Bid* mRNA expression after OGD/R. The data are expressed as the means±S.E.M. from three independent experiments. *P<0.05 *versus* control, ^#^*P*<0.05 *versus* OGD/R and ^&^*P*<0.05 *versus* ANXA1+OGD/R. (**b**) Western blot results showing the effects of ANXA1 overexpression and IPZ on Bid protein expression after OGD/R. (**c**) Statistical analysis of the data shown in (**b**). The data are expressed as the means±S.E.M. from three independent experiments. *P<0.05 *versus* control, ^#^*P*<0.05 *versus* OGD/R and ^&^*P*<0.05 *versus* ANXA1+OGD/R. (**d**) qPCR results showing the expression of *Bid* mRNA after treatment with *ANXA1* shRNA. The data are expressed as the means±S.E.M. from three independent experiments. *P<0.05 *versus* Scr and ^#^*P*<0.05 *versus* Scr+OGD/R. (**e**) Western blot showing expression of the Bid protein after treatment with *ANXA1* shRNA. (**f**) Statistical analysis of the data shown in (**e**). The data are expressed as the means±S.E.M. from three independent experiments. *P<0.05 *versus* Scr and ^#^*P*<0.05 *versus* Scr+OGD/R

To examine the function of ANXA1 on *Bid* expression, we treated primary cultured neurons and HEK293 cells with IPZ to decrease nuclear ANXA1 translocation and then examined *Bid* expression. As shown in Figure 3a, quantitative real-time PCR (qPCR) demonstrated that the upregulation of ANXA1 after OGD/R increased *Bid* expression, but treatment with IPZ downregulated the expression of *Bid*. Similar trends were observed for protein levels (Figures 3b and c). To block the expression of ANXA1 in primary cultured neurons, we constructed an adenoviral vector that encodes short hairpin RNA (shRNA) against rat *ANXA1*. Western blot analysis showed that treatment with shRNA resulted in observable knockdown (Supplementary Figures S2a and b). Then, we examined the expression of Bid. Downregulation of ANXA1 resulted in markedly decreased of *Bid* expression in primary cultured neurons under OGD/R conditions (Figures 3d–f). In addition, we used human *ANXA1* shRNAs to knock down *ANXA1* expression in HEK293 cells. As shown in Supplementary Figures S3a and b, the no. 2 shRNA against *ANXA1* efficiently decreased ANXA1 expression. Then, we examined the expression of Bid. Silencing *ANXA1* with shRNA no. 2 decreased *Bid* expression under OGD/R conditions at both the mRNA and protein level in HEK293 cells (Figures 3d–f).

### ANXA1 interacts with p53 in the nucleus following OGD/R

**Figure 4:**
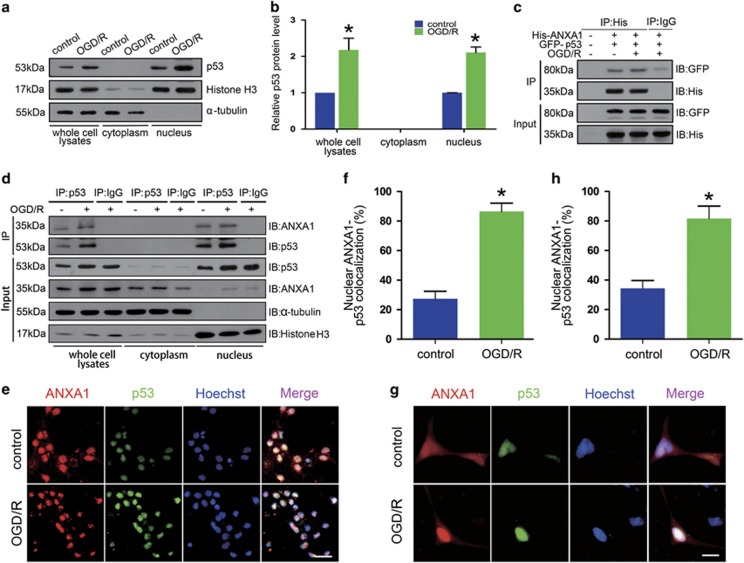
ANXA1 interacts with p53 in the nucleus after OGD/R. (**a**) Western blot results demonstrating the expression of p53 in the cytoplasm and nucleus in primary cultured neurons treated without or with OGD/R. (**b**) Quantitative densitometry analysis of the p53 expression shown in (**a**). The data are expressed as the means±S.E.M. from three independent experiments. *P<0.05 *versus* control. (**c**) Representative Co-IP results show the interactions of ANXA1 with p53 in HEK293 cells transfected with pcDNA-ANXA1 (His-tagged) and pEGFP-p53 (GFP-tagged) treated without or with OGD/R. (**d**) Representative Co-IP results show the interactions of ANXA1 with p53 in primary cultured neurons treated without or with OGD/R. (**e**) Immunofluorescence shows the colocalization of endogenous p53 and ANXA1 in primary cultured neurons treated with or without OGD/R. Scale bar=50 *μ*m. (**f**) The nuclear colocalization of endogenous ANXA1 and p53 was quantitated as a graph. The data are expressed as the means±S.E.M. from three independent experiments. *P<0.05 *versus* control. (**g**) Immunofluorescence shows the colocalization of ANXA1 and p53 in HEK293 cells transfected with pjRed-ANXA1 and pEGFP-p53 treated with or without OGD/R. Scale bar=50 *μ*m. (**h**) The nuclear colocalization of endogenous ANXA1 and p53 was quantitated as a graph. The data are expressed as the means±S.E.M. from three independent experiments. *P<0.05 *versus* control

The p53 protein has been reported to function as a *Bid* gene transcription factor. To examine the role of p53, we first determined the subcellular location of p53 in primary cultured neurons following OGD/R. We found that OGD/R increased the expression of p53 in whole-cell lysates and promoted its nuclear accumulation (Figures 4a and b). We then constructed the plasmids ANXA1-His and p53-GFP (green fluorescent protein) to assess the relationship between ANXA1 and p53 in HEK293 cells. Co-immunoprecipitation (Co-IP) results revealed that ANXA1 associated with p53 (Figure 4c). In addition, we examined the subcellular location of the interaction in primary cultured neurons, which suggested that OGD/R increased ANXA1–p53 interactions and that this interaction occurred primarily in the nucleus (Figure 4d). Immunofluorescence analysis also suggested that in primary cultured neurons, endogenous ANXA1 colocalized with p53 in the nucleus after OGD/R (Figures 4e and f). In addition, we transfected the plasmids pjRed-ANXA1 (red) and pEGFP-p53 (green) into HEK293 cells to confirm the interaction by immunofluorescence. These results also indicated that OGD/R increased the interaction between ANXA1 and p53 in the nucleus (Figures 4g and h).

### ANXA1 regulates p53 transcriptional activation after OGD/R

**Figure 5:**
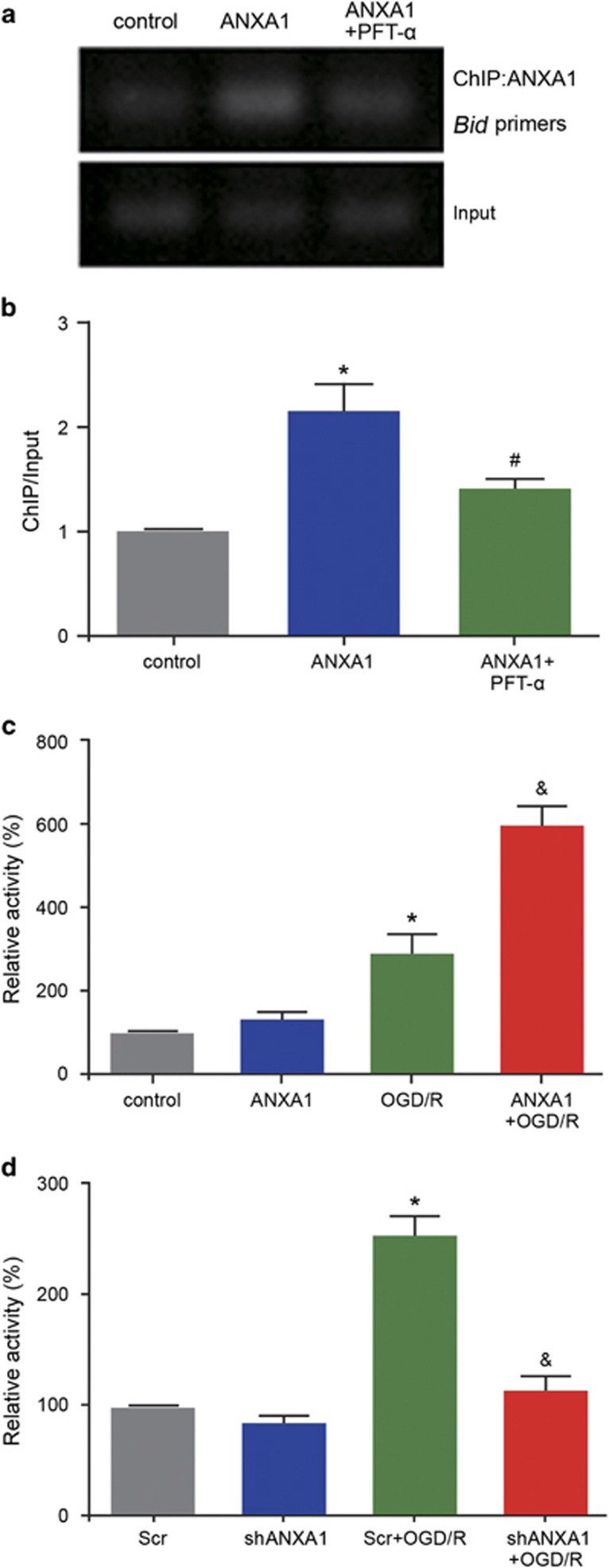
Effect of ANXA1 on p53 transcriptional activity. (**a**) ChIP-PCR analysis showing the combination of ANXA1 with *Bid* following treatment of HEK293 cells with ANXA1 overexpression and PFT-*α*. (**b**) Statistical analysis of the data shown in (**a**). The data are expressed as the means±S.E.M. from three independent experiments. *P<0.05 *versus* control and ^#^*P*<0.05 *versus* ANXA1. (**c**) The luciferase assays show the transcriptional activity of p53 following treatment with ANXA1 after OGD/R in HEK293 cells. The data are expressed as the means±S.E.M. from three independent experiments. *P<0.05 *versus* control and ^#^*P*<0.05 *versus* OGD/R. (**d**) The luciferase assays show the transcriptional activity of p53 following treatment with *ANXA1* shRNA in HEK293 cells. The data are expressed as the means±S.E.M. from three independent experiments. *P<0.05 *versus* Scr and ^&^*P*<0.05 *versus* Scr+OGD/R

To examine the function of the ANXA1–p53 interaction, we treated HEK293 cells with pifithrin-*α* (PFT-*α*) (20 *μ*M) to inhibit the function of p53. ChIP-PCR was used to assess the interaction between ANXA1 and the *Bid* gene promoter. The results revealed that the interaction between ANXA1 and the *Bid* promoter was inhibited by PFT-*α* (Figures 5a and b). A luciferase reporter assay was then conducted to examine p53 transcriptional activity in HEK293 cells, which suggested that compared with normal conditions, OGD/R increased p53 transcriptional activation, and that compared with OGD/R alone, ANXA1 overexpression+OGD/R significantly increased p53 transcriptional activation (*P*<0.05; Figure 5c). In contrast, shRNA-mediated ANXA1 silencing demonstrated that compared with OGD/R+scrambled control DNA (Scr), OGD/R+*ANXA1* shRNA decreased p53 transcriptional activation (Figure 5d). Taken together, these results suggested that ANXA1 regulated p53 transcriptional activation and that the binding between ANXA1 and the *Bid* gene promoter could be blocked by p53 inhibition.

### ANXA1 coregulates Bid with p53

**Figure 6:**
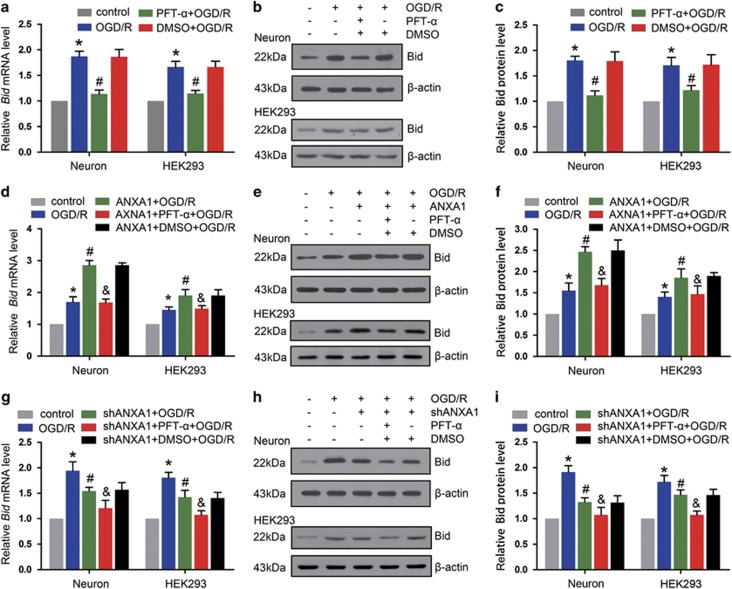
ANXA1 coregulates the expression of Bid with p53. (**a**) qPCR results showing the expression of *Bid* mRNA after treatment with PFT-*α*. The data are expressed as the means±S.E.M. from three independent experiments. *P<0.05 *versus* control and ^#^*P*<0.05 *versus* OGD/R. (**b**) Western blot showing expression of the Bid protein after treatment with PFT-*α* in primary cultured neurons or HEK293 cells. (**c**) Statistical analysis of the data shown in (**b**). The data are expressed as the means±S.E.M. from three independent experiments. *P<0.05 *versus* control and ^#^*P*<0.05 *versus* OGD/R. (**d**) Statistical analysis of qPCR results showing the expression of *Bid* mRNA after treatment with ANXA1 and PFT-*α*. The data are expressed as the means±S.E.M. from three independent experiments. *P<0.05 *versus* control, ^#^*P*<0.05 *versus* OGD/R and ^&^*P*<0.05 *versus* ANXA1+OGD/R. (**e**) Western blot showing expression of the Bid protein after treatment with ANXA1 and PFT-*α* in primary cultured neurons or HEK293 cells. (**f**) Statistical analysis of the data shown in (**e**). The data are expressed as the means±S.E.M. from three independent experiments. *P<0.05 *versus* control, ^#^*P*<0.05 *versus* OGD/R and ^&^*P*<0.05 *versus* ANXA1+OGD/R. (**g**) Statistical analysis of the qPCR results shows the expression of *Bid* mRNA after treatment with *ANXA1* shRNA and PFT-*α*. The data are expressed as the means±S.E.M. from three independent experiments. *P<0.05 *versus* control, ^#^*P*<0.05 *versus* OGD/R and ^&^*P*<0.05 *versus ANXA1* shRNA+OGD/R. (**h**) Western blot showing expression of the Bid protein after treatment with *ANXA1* shRNA and PFT-*α*. (**i**) Statistical analysis of the data shown in (**h**). The data are expressed as the means±S.E.M. from three independent experiments. *P<0.05 *versus* control, ^#^*P*<0.05 *versus* OGD/R and ^&^*P*<0.05 *versus ANXA1* shRNA+OGD/R

Despite demonstrating their association and DNA binding, the function of the ANXA1–p53 interaction remained unclear. To address this issue, we examined the expression of Bid using qPCR and western blot analysis. As shown in Figure 6a, OGD/R significantly increased *Bid* expression, whereas PFT-*α* markedly decreased the expression of *Bid* mRNA. Similar results were observed by western blot in neuron analysis (Figures 6b and c). These results were also confirmed in HEK293 cells (Figures 6a–c). In addition, we found that the upregulation of Bid induced by ANXA1 overexpression was reversed by PFT-*α* under OGD/R conditions (Figures 6d–f). Finally, we demonstrated that along with ANXA1 downregulated by used *ANXA1* shRNA, the level of *Bid* expression was also decreased after OGD/R. Treatment with *ANXA1* shRNA+PFT-*α* showed that the level of *Bid* expression was lower compared with when treated with *ANXA1* shRNA after OGD/R in primary cultured neurons and HEK293 cells (Figures 6g–i).

### ANXA1 regulates cell apoptosis via the caspase-3 pathway

**Figure 7:**
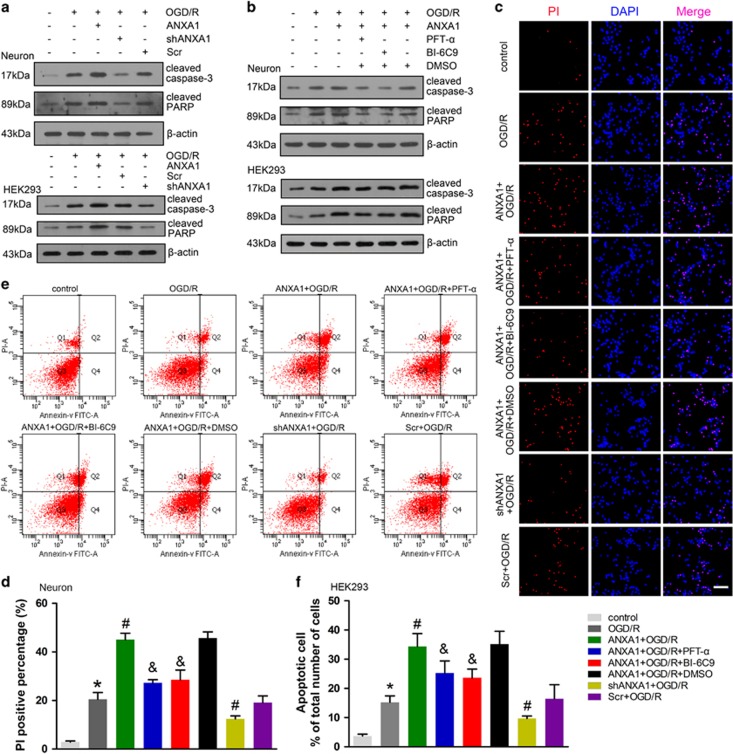
Effect of ANXA1 on the caspase-3 apoptotic pathway. (**a**) Western blot showing the expression of cleaved caspase-3 and PARP following treatment with ANXA1 or *ANXA1* shRNA in neuronal cells and HEK293 cells. The results were obtained in three independent experiments. (**b**) Western blot showing the expression of cleaved caspase-3 and PARP following treatment with ANXA1, PFT-*α*, or BI-6C9 in primary cultured neurons or HEK293 cells. The results were obtained in three independent experiments. (**c**) PI staining results demonstrating cell death following treatment with ANXA1, PFT-*α*, BI-6C9, or *ANXA1* shRNA in primary cultured neurons. Scale bar=50 *μ*m. (**d**) Statistical analysis of the data shown in (**c**). The data are expressed as the means±S.E.M. from three independent experiments. *P<0.05 *versus* control, ^#^*P*<0.05 *versus* OGD/R and ^&^*P*<0.05 *versus* ANXA1+OGD/R. (**e**) Flow cytometry results demonstrating apoptosis following treatment with ANXA1, PFT-*α*, BI-6C9, or *ANXA1* shRNA in HEK293 cells. (**f**) Statistical analysis of the data shown in (**e**). The data are expressed as the means±S.E.M. from three independent experiments. *P<0.05 *versus* control, ^#^*P*<0.05 *versus* OGD/R and ^&^*P*<0.05 *versus* ANXA1+OGD/R

Previous data have indicated that nuclear ANXA1 migration upregulates *Bid* expression, which is involved in OGD/R-induced cell apoptosis. However, the exact apoptosis pathway involved in this process had not been determined. The caspase-3-PARP is a classic apoptosis pathway. We found that ANXA1 overexpression increased the expression of cleaved caspase-3 and PARP in neuron and that this increase was reduced by *ANXA1* shRNA following OGD/R (Figure 7a). In addition, ANXA1-induced caspase-3 activation was downregulated by treatment with PFT-*α* (20 *μ*M) or the Bid inhibitor BI-6C9 (2 *μ*M) in primary cultured neurons (Figure 7b). These results were also confirmed in HEK293 cells (Figures 7a and b). Taken together, these data indicated that ANXA1 regulated caspase-3 activation via the p53-Bid pathway under OGD/R conditions. Similar trends were observed by PI staining, and the results indicated that ANXA1-induced cell apoptosis was impeded by treatment with PFT-*α*, BI-6C9, or *ANXA1* shRNA in primary cultured neurons (Figures 7c and d). These results were also confirmed in HEK293 cells by flow cytometry (Figures 7e and f).

## Discussion

In the present study, we elucidated the role of nuclear ANXA1 migration in OGD/R-induced cell apoptosis. Specifically, we found that following OGD/R, ANXA1 translocated from the cytoplasm to the nucleus and that this migration could be blocked by the Imp*β* inhibitor IPZ. In the nucleus, ANXA1 interacted with the transcription factor p53. Conditions of OGD/R+ANXA1 increased the transcriptional activity of p53, thereby increasing *Bid* expression. This process was inhibited by the p53 inhibitor, PFT-*α*. Notably, we found that silencing ANXA1 with shRNA could also decrease *Bid* expression and enhance cell survival after OGD/R.

The first part of this study was aimed to address the mechanism by which ANXA1 influences OGD/R-induced cell apoptosis. We found that OGD/R not only increased ANXA1 expression in whole-cell lysates but also increased the accumulation of ANXA1 in the nucleus. Previous data indicated that the nuclear translocation of ANXA1 was due to stimulation by the mitogen PMA. PMA induced the expression and phosphorylation of ANXA1, which then increased ANXA1 nuclear migration.^12^ As OGD/R stimulation activates TRPM7 function and ANXA1 has been shown to be phosphorylated by the TRPM7 kinase,^31, 32^ we speculated that ANXA1 upregulation was due to TRPM7 kinase activation following OGD/R. Consistent with this possibility, our previous data revealed that truncation of the TRPM7 kinase decreased nuclear ANXA1 translocation.^9^ In addition, we demonstrated that the decreased cell-survival rate observed after OGD/R was due to nuclear ANXA1 migration.

The full-length ANXA1 consists of 346 amino acids and contains an N-terminal domain and a C-terminal core domain. The central domain of ANXA1 is composed of four repeat sequences.^33, 34^ Previous studies have provided evidence that the ANXA1-dependent inhibition of local and systemic inflammatory processes could be recapitulated using the ANXA1 mimetic peptide Ac 2–26, which comprises the first 25 amino acids of ANXA1.^35, 36, 37^ In this study, we found that the nuclear translocation of overexpressed ANXA1 might increase neuronal cells death after OGD/R, and we speculate that ANXA1-induced cell death was due to its remaining amino-acid sequence. Considering that ANXA1 lacks a classical nuclear localization signal, we further speculate that nuclear ANXA1 translocation was due to a non-classical translocation pathway. In this study, we also found that the classic nuclear-migration pathway inhibitor IMT had no effect on ANXA1 nuclear translocation, but that the non-classical-migration pathway inhibitor IPZ, which blocked the function of Imp*β*, decreased nuclear ANXA1 accumulation. Identification of the specific amino-acid sequences that perform key roles in regulating nuclear ANXA1 translocation and cell death induction remain to be determined in future studies.

We next investigated ANXA1 function in the nucleus using ChIP-Seq analysis. We immunoprecipitated ANXA1-DNA complexes with an ANXA1 antibody and then determined the bound DNA components using high-throughput sequencing. GO analysis indicated that ANXA1 might be involved in various cellular processes, including cell apoptosis. Considering that ANXA1 does not contain a DNA-binding element, we speculate that ANXA1 might associate with transcription factors to regulate gene expression. Consistent with this hypothesis, we found that ANXA1 interacted with p53, which is well known as a *Bid* gene transcription factor,^16^ and our findings demonstrated that ANXA1 increased p53-dependent transcriptional activation. These data indicated that ANXA1 interacted with p53 and enhanced transcriptional activation and that this complex regulated *Bid* expression.

**Figure 8:**
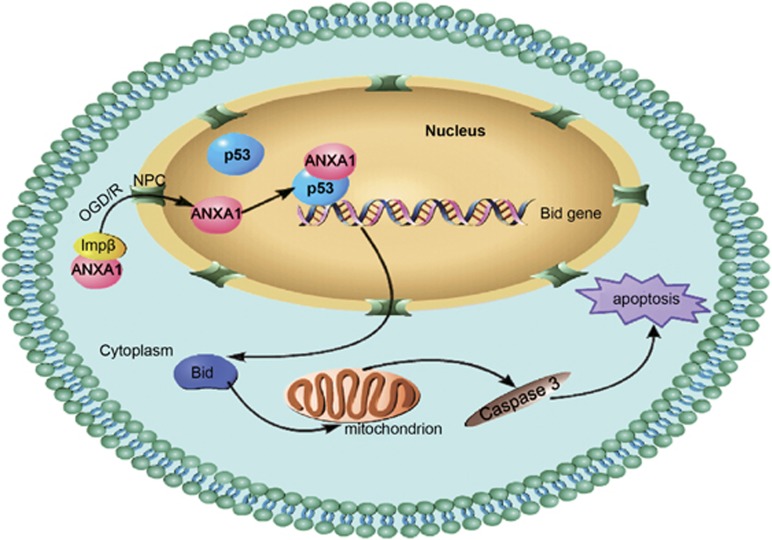
Schematic representation of the contribution of ANXA1 to apoptosis after OGD/R. Nuclear ANXA1 translocation is mediated by Imp*β* function after OGD/R and induces apoptosis via the p53-Bid-caspase-3 pathway

Taken together, these data suggested that the non-classical mechanism by which ANXA1 is translocated into the nucleus involves transport by Imp*β* function. In addition, the results contribute to our knowledge of ANXA1 by elucidating the function of its nuclear migration after OGD/R. This study is the first to demonstrate that ANXA1 interacts with p53 to coregulate *Bid* expression and induce cell death after OGD/R via the caspase-3 pathway (Figure 8). Collectively, these observations provide a more comprehensive understanding of ANXA1 function in cell apoptosis after OGD/R and strongly suggest that decreasing nuclear ANXA1 migration might be a useful therapeutic approach for treating ischemic stroke.

## Materials and Methods

### Reagents and antibodies

IPZ was obtained from Calbiochem (Darmstadt, Germany; 401105). IMT, PFT-*α*, and BI-6C9 were obtained from Sigma-Aldrich China (Shanghai, China; I8898, P4236, and B0186, respectively). The following antibodies were used: anti-annexin 1 (Santa Cruz, Dallas, TX, USA; sc-11387, 1 : 1000), anti-histone H3 (Cell Signaling Technology, Beverly, MA, USA; no. 4499, 1 : 1000), anti-p53 (Santa Cruz; sc-126, 1 : 1000), anti-cleaved caspase-3 (Cell Signaling Technology; no. 9664, 1 : 1000), anti-cleaved PARP (Cell Signaling Technology; no. 5625, 1 : 1000), anti-GFP (Santa Cruz; sc-9996, 1 : 1000), anti-His (Santa Cruz; sc-803, 1 : 1000), anti-*α*-tubulin (Santa Cruz; sc-53646, 1 : 1000), and anti-*β*-actin (Santa Cruz; sc-47778, 1 : 1000).

### Cell culture

Primary cultured rat cerebral cortical neurons were prepared from 16- to 18- day-old Sprague–Dawley rat embryos, as described previously.^38^ The protocol for the use of rats for neuronal cultures was performed according to the principles of the Animal Care Committee of Huazhong University of Science and Technology. Briefly, rat embryos were decapitated and the tissues of the cerebral cortex were isolated under a dissection microscope, cut into ~1-mm^3^ pieces, and incubated with 0.25% trypsin-EDTA for 15 min at 37 °C. Freshly prepared Dulbecco's modified Eagle's medium: nutrient mixture F12 (DMEM-F12) medium containing 10% fetal bovine serum (FBS; Gibco, Gaithersburg, MD, USA) was used to stop the trypsin digestion. Subsequently, fire-polished glass pipettes were used to gently triturate the tissue mass into a cell suspension. Finally, the cells were counted and plated in poly-L-lysine-coated culture dishes or coverslips in 24-well plates at a density of 1 × 10^6^ cells per dish or 2 × 10^5^ cells per well, respectively. Neurons were maintained at 37 °C in a humidified atmosphere containing 5% CO_2_. After 24 h, the culture medium was replaced with Neurobasal Medium supplemented with 2% B-27, and the cultures were fed two times per week. Neurons were used for the experiments between days 7 and 10 *in vitro*.

HEK293 cells were maintained in DMEM supplemented with 10% FBS (Gibco), penicillin (100 U/ml)/streptomycin (100 *μ*g/ml) and 2 mM L-glutamine at 37 °C in a 5% CO_2_. Confluent cell layers were split two times per week. Transfections were performed using Lipofectamine 2000 (Invitrogen) when the cells were 80–90% confluent.

### Plasmids

DNA fragments corresponding to the full-length ANXA1 and p53 coding sequences were amplified by PCR, followed by cloning into the pcDNA3.0 plasmid or pEGFP-N1 vector (Invitrogen). To express ANXA1 tagged with red fluorescence protein, the pjRED-C1-ANXA1 plasmid was constructed by PCR amplification of the ANXA1 coding sequence, followed by cloning into the pjRed-C1 vector (gift from Professor He Li, HUST, China). Human *ANXA1* shRNA plasmids were purchased from GenePharma (Suzhou, Wuhan, China). The target sequence for *ANXA1* (GenBank No. NM_000700.2) shRNA no. 1 was 5′-AGCTTGAGACCATCAAGGG-3′, and the target sequence for *ANXA1* shRNA no. 2 was 5′-AGAACAACTTGTATAGGGT-3′.

### Adenoviral infection

The adenoviral vectors carried GFP, rat *ANXA1* and *ANXA1* shRNA or the Scr were constructed by Vigene Biosciences Co. Ltd. (Shandong, China). The shRNA sequence that targets rat *ANXA1* sequence (GenBank No. NM_012904.2) was designed as follows: 5′-GCCTCACAACCATTGTGAAGT-3′, and a Scr shRNA served as a negative control. Primary cultured neurons were infected with *ANXA1*, *ANXA1* shRNA, or the Scr adenoviral particles. The optimal multiplicity of infection was determined to be 50 : 1 to 100 : 1, based on the observed fluorescence intensity of GFP. After the adenovirus infection for 48 h, cells were subjected to OGD/R and (or) other treatments.

### Establishment of the OGD/R model

The cell culture medium was replaced with glucose-free DMEM equilibrated with nitrogen, after which neuron and HEK293 cells were transferred to an incubator containing 5% CO_2_ and 95% N_2_ at 37 °C for 1 h. After washing the cultures with DMEM three times, the cultures were maintained in glucose-containing normoxic DMEM at 37 °C in a humidified 5% CO_2_ incubator. In all experiments, the culture medium pH was maintained at 7.2.

### Protein extraction and preparation

Subcellular fractionation was performed as described previously.^9^ Cell pellets were resuspended with buffer A (40 mM Tris-HCl, 10 mM NaCl, 1 mM EDTA, 1 mM DTT, and protease inhibitors). Resuspended cells were incubated on ice for 15 min and mixed by vortexing every 5 min for 5 s. Then, 30 *μ*l 10% NP-40 was added to the cell extracts and vigorously shaken for 10 s. After centrifugation at 14 000 × *g* for 10 min at 4 °C, the supernatants were transferred to a new tube (the cytosolic fraction). The pellets were resuspended with buffer B (40 mM Tris-HCl, 420 mM NaCl, 10% glycerol, 1 mM EDTA, 1 mM DTT, and protease inhibitors). The resuspended extracts were incubated on ice for 20 min and mixed vigorously by vortexing every 5 min for 5 s. After centrifugation at 14 000 × *g* for 10 min at 4 °C, the supernatants were transferred to a new tube (the nuclear fraction).

### Western blot analysis

Proteins were run on 10 or 12% polyacrylamide gels and transferred to polyvinylidene difluoride (PVDF) membranes. PVDF membranes were blocked with 5% bovine serum albumin at room temperature for 60–90 min and incubated overnight at 4 °C with antigen-specific primary antibodies. Blots were then incubated with species-specific HRP-conjugated secondary antibodies for 60 min at room temperature. Proteins were visualized by incubation with a Chemiluminescence Substrate Kit (ECL Plus; Perkin-Elmer Inc., Covina, CA, USA). The expression of the target proteins was quantified using the ImageJ software (NIH, Bethesda, MD, USA) after normalizing to *β*-actin, *α*-tubulin, or histone H3 expression.

### Co-IP

CO-IP was performed as described previously.^7^ Briefly, cell lysates were generated by sonication in a buffer containing 20 mM HEPES, 400 mM KCl, 5% glycerol, 5 mM EDTA, 0.4% NP-40, and protease inhibitors, and precleared by centrifugation. The cell lysates were then incubated with an anti-GFP or anti-p53 antibody overnight at 4 °C. The reaction mixture was then incubated with protein A/G PLUS-Agarose beads (Santa Cruz; sc-2003) for 2 h at 4 °C. The precipitates were washed three times with wash buffer and then eluted from the protein A/G PLUS-Agarose beads by boiling with 1 × sodium dodecyl sulfate (SDS) for 5 min at 95 °C. The protein samples were resolved by SDS-polyacrylamide gel electrophoresis.

### Immunofluorescence

Indirect immunofluorescence analysis was performed as described previously.^9^ The cultured neurons (fixed in 4% paraformaldehyde) were gently washed two times with PBS preheated to 37 °C. The cells were then subjected to treatment with 10% Triton X-100 for 10 min to rupture the cell membranes. The fixed cells were again washed with PBS for 15 min, and the cells were again treated with 5% bovine serum albumin for another 40 min to block nonspecific binding. The cells were then incubated with a rabbit polyclonal ANXA1 antibody (1 : 200) or a mouse monoclonal p53 antibody (1 : 200) in 5% bovine serum albumin at 4 °C overnight. The cells were washed and subsequently incubated with secondary antibody in 5% bovine serum albumin at 37 °C for 1 h. A Zeiss double-photon fluorescence microscope (Zeiss 510 Meta, Oberkochen, Germany) was used to detect the fluorescence.

### Quantitative real-time PCR

Cells were transfected with or without ANXA1, *ANXA1* shRNA or Scr. After transfection, total RNA was prepared with the TRIzol reagent (Invitrogen), and cDNA was synthesized from 1 *μ*g RNA using the ReverTra Ace-*α*-TM First Strand cDNA Synthesis Kit (Toyobo, Osaka, Japan). qPCR was performed with SYBR Green Real-Time PCR Master Mix (Toyobo) on a C1000 Thermal Cycler (Bio-Rad Laboratories, Hercules, CA, USA), according to the manufacturer's recommendations. The primers used were as follows: rat *Bid*, 5′-CGACGAGGTGAAGACATCCT-3′ (forward primer) and 5′-AGCAGAGATGGTGCATGACT-3′ (reverse primer); human *Bid*, 5′-ACTGGTGTTTGGCTTCCTCC-3′ (forward primer) and 5′-ATTCTTCCCAAGCGGGAGTG-3′ (reverse primer); *rat β-actin*, 5′-TAAGGCCAACCGTGAAAAGAT-3′ (forward primer) and 5′-GGTACGACCAGAGGCATACA-3′ (reverse primer). Human *β-actin*, 5′-TCCACCACCCTGTTGCTGTA-3′ (forward primer) and 5′-ACCACAGTCCATGCCATCAC-3′ (reverse primer).

### Luciferase reporter assay

Cells were routinely co-transfected with a TK-*Renilla* luciferase plasmid (Promega, Madison, WI, USA) to normalize for the transfection efficacy. To determine luciferase expression, the pGL4.38 [luc2P/p53 RE/Hygro] Vector (Promega) was transfected into HEK293 cells 1 day after plating. The indicated treatments were performed 24 h after transfection, and the cells were collected according to the manufacturer's instructions (Roche Diagnostics, Roswell, GA, USA). Briefly, the cell lysates were prepared with 1 × lysis buffer, and the luciferase reporter signals were detected immediately after adding luciferin substrates using a Fluoroskan Ascent FL System (Thermo Scientific, Waltham, MA, USA). The data shown represent the mean values obtained from three independent experiments.

### Flow cytometry

The effects of ANXA1 on apoptosis were evaluated using a FITC-Annexin V Apoptosis Detection Kit (BD Pharmingen, San Diego, CA, USA). Briefly, 10^5^ cells were harvested through trypsinization and washed two times with cold PBS. The cells were then centrifuged at 1000 r.p.m. for 5 min, and the supernatant was discarded and the pellet was resuspended in 500 *μ*l 1 × Annexin V-binding buffer in a 1.5-ml culture tube and later incubated with 5 *μ*l of an FITC-conjugated Annexin V and 5 *μ*l PI for 10 min at room temperature in the dark. The samples were analyzed by fluorescence-activated cell sorting using the Cell Quest Research software (Beckman Coulter, Brea, CA, USA).

### Statistical analysis

Data are expressed as the mean value±S.E.M. Statistical significance was calculated using the Student's *t*-test. A value of *P*<0.05 was considered statistically significant. All results shown are representative of at least three independent experiments.

## Abbreviations

ANXA1: annexin 1
OGD/R: oxygen-glucose deprivation–reoxygenation
Co-IP: co-immunoprecipitation
ChIP: chromatin immunoprecipitation assay
IPZ: importazole
IMT: ivermectin
shRNA: short hairpin RNA
Scr: scrambled control DNA
PARP: poly(ADP-ribose) polymerase
PFT-*α*: pifithrin-*α*
PMA: phorbol 12-myristate 13-acetate
Imp*β*: importin *β*
PVDF: polyvinylidene difluoride
