# Supplementary material for: Nuclear translocation of annexin 1 following oxygen-glucose deprivation–reperfusion induces apoptosis by regulating Bid expression via p53 binding
Source: Cell Death Dis. 2016 Sep 1;7(9):e2356–. doi: 10.1038/cddis.2016.259 (PMC5059862; doi:10.1038/cddis.2016.259)
Supplement: Supplementary Information [file cddis2016259x1.doc]

**Supplementary Information for:**

**Nuclear translocation of annexin 1 following oxygen-glucose deprivation/reperfusion induces apoptosis by regulating Bid expression via p53 binding**

**Supplementary Figure Legends:**

**Supplementary Figure S1:** ANXA1 protein levels increased following transfection with ANXA1 adenoviral particles in primary cultured neurons. (**a**) The purity of adherent cells was verified by neuron specific marker NeuN. Scale bars =50 μm. (**b**) Western blot analysis of ANXA1 expression in primary cultured neurons transfected with ANXA1 at 24, 48 and 72 h, respectively. (**c**) Statistical analysis of the data shown in Supplementary Figure S1b. The data are expressed as the means ± SEM from three independent experiments. *P < 0.01 versus control.

**Supplementary Figure S2:** Adenoviral transfection of *ANXA1* shRNA reduces ANXA1 expression. (**a**) Primary cultured neurons were transfected with either Scr or *ANXA1* shRNA adenoviral particles for 48 h. Western blot were performed to examine the blocking efficiency. (**b**) Statistical analysis of the data shown in Supplementary Figure S2a. The data are expressed as the means ± SEM from three independent experiments. *P < 0.05 versus control.

**Supplementary Figure S3:** Effective shRNA-mediated suppression of the ANXA1 expression.

(**a**) HEK293 cells were transfected with shRNA plasmid expressing Scr, ANXA1-targeting shRNA#1, ANXA1-targeting shRNA#2. The knockdown of endogenous ANXA1 expression was confirmed by western blot analysis. (**b**) Statistical analysis of the data shown in Supplementary Figure S3a. The data are expressed as the means ± SEM from three independent experiments. *P < 0.01 versus control.

**Supplementary Materials and Methods**

**Chromatin Immunoprecipitation (ChIP)**: The ChIP-Seq procedure was performed as previously described. Briefly, primary cultured neurons or HEK293 cells (1 × 10^7^ cells/10 cm petri dish) were cross-linked directly on plates by adding 37% formaldehyde to the media (final concentration, 1.4%) for 15 min at room temperature. Cross-linking was stopped by adding glycine (final concentration, 125 mM), followed by incubation for 5 min at room temperature. Cells were scraped off in 500 μl of ice-cold 2× phosphate-buffered saline (PBS) and transferred to Eppendorf tubes and placed on ice. Cells were centrifuged at 2 000 × *g* for 5 min at 4°C and washed twice with ice-cold PBS. Cells were lysed in 500 μl of ice-cold immunoprecipitation buffer (150 mM NaCl, 50 mM Tris–HCl [pH 7.5], 5 mM EDTA, 1% Triton X-100, 0.5% NP40) supplemented with Complete Mini Protease Inhibitor Cocktail (Roche), hereafter referred to as “supplemented IP-Buffer.” Samples were centrifuged at 12 000 × *g* for 1 min at 4°C and washed once in ice-cold supplemented IP-Buffer. Five hundred microliters of ice-cold supplemented IP-Buffer was added to the pelleted nuclei and the samples were re-suspended. Chromatin was fragmented by sonication using a BioRuptor (Diagenode; 80 cycles, 30 s on and 30 s off) to generate fragments of 150–300 bp. Fragment lengths were examined by agarose gel electrophoresis. Samples were centrifuged at 12 000 × *g* for 10 min at 4°C. Supernatants were transferred to fresh ice-cold tubes and stored at −80°C. Samples were diluted with an equal volume of D-Buffer (150 mM NaCl, 50 mM Tris–HCl [pH 7.5], 5 mM EDTA, 1% Triton X-100). Five micrograms of an anti-ANXA1 antibody (Santa Cruz, sc-11387) was used for each ChIP experiment. Samples were incubated in an ultrasonic water bath for 25 min at 4°C and then centrifuged at 12 000 × *g* for 10 min at 4°C. Twenty microliters of magnetic AG beads (Invitrogen) were used for each ChIP experiment. Beads were washed 3 times in 1 ml of supplemented IP-Buffer before use. After the final wash, beads were suspended in 40 μl of supplemented IP-Buffer and mixed with 90% of the chromatin supernatant described above. The mix was incubated overnight at 4°C, with rotation. The beads were washed twice with 200 μl of ice-cold supplemented IP Buffer and then 3 times with 200 μl ice-cold D-Buffer supplemented with protease inhibitors. Cross-linking was reversed by adding Reverse X-link Buffer and protease K (Invitrogen), with incubation in a water bath for 20 min at 55°C. The remaining 10% of the reversed cross-linking chromatin supernatant was used as input. Supernatants were transferred to fresh tubes and incubated at 95°C to inactivate the protease K. DNA was purified using DNA-Purification Buffer and DNA-Purification Magnetic Beads, as recommended by the manufacturer (Invitrogen). The primers used for PCR to detect the human *Bid* gene were as follows: 5'-CCT GGG TGA GTA TCT GGA ATG-3' (forward primer) and 5'-CTG GAA AGG GAC ACA CAG AGT-3' (reverse primer).

**ChIP-Seq.** Primary cultured rat neurons were used for ChIP-Seq analysis. DNA was sequenced using the Illumina HiSeq 2000 platform at BGI in Shenzhen, China. Library preparation, cluster generation, and sequencing by synthesis were performed according to manufacturer’s protocol. All raw reads were aligned using Burrows–Wheeler Aligner software (BWA version 0.5.8c [r1536]) and the rat reference genome. Aligned reads were processed by model-based analysis of ChIP-Seq (MACS) 1.4.0 rc2 for peak calling. Significant peaks were defined using the criteria of a threshold of a minimum of 9 reads and a p value of less than 10^−5^, as suggested previously. Input ChIP DNA was used as negative control. ChIP experiments were independently repeated, and ChIP-Seq-called peaks were verified by qPCR. We normalized the rat tags. We used the median of the total number of aligned tags across all the samples. We used this median as a reference total count. We then scaled bin counts in all samples to obtain a new total sample count equal to the reference total count. The typical reference total count was 150 million tags for rat. The input samples were normalized to the same total reference count as the ChIP samples.

1. Joel R, Ghia E, Auerbach RK, Zhang ZD, Theodore G, Robert B*, et al.* PeakSeq enables systematic scoring of ChIP-seq experiments relative to controls. *Nat Biotechnol* 2009, **27**(1)**:** 66-75.

2. Rodier G, Kirsh O, Baraibar M, Houles T, Lacroix M, Delpech H*, et al.* The transcription factor E4F1 coordinates CHK1-dependent checkpoint and mitochondrial functions. *Cell reports* 2015, **11**(2)**:** 220-233.

3. Luo Y, Blechingberg J, Fernandes AM, Li S, Fryland T, Borglum AD*, et al.* EWS and FUS bind a subset of transcribed genes encoding proteins enriched in RNA regulatory functions. *Bmc Genomics* 2015, **16**(1)**:** 929.

4. Taslim C, Huang K, Huang T, Lin S. Analyzing ChIP-seq Data: Preprocessing, Normalization, Differential Identification, and Binding Pattern Characterization. In: Wang J, Tan CA, Tian T (eds). *Next Generation Microarray Bioinformatics: Methods and Protocols*. Humana Press: Totowa, NJ, 2012, pp 275-291.
